# Comparative evaluation on the effect of different cavity disinfectant nano gels; Chlorohexidine, Propolis, Liquorice versus Diode Laser in terms of composite microleakage (comparative in vitro study)

**DOI:** 10.1038/s41405-023-00176-2

**Published:** 2023-11-11

**Authors:** Maryam Mohamed ElMansy, Silvia Sabry Tawfik Tadros, Reham Sayed Saleh, Rehab Abdelmonem, Hala El Menoufy, Naglaa Shawky

**Affiliations:** 1 Researcher of Pediatric Dentistry, Orthodontic and Pediatric Dentistry Department, Orodental Institute, National Research Centre, Dokki, Giza, Egypt; 2 Lecturer of Conservative Dentistry, Conservative Surgery Department, College of Oral and Dental Surgery, Misr University for Science and Technology(MUST), 6th of October City, Giza, Egypt; 3 Researcher of Restorative Dentistry, Restorative and Dental Materials Department, Orodental Institute, National Research Centre, Dokki, Giza, Egypt; 4 Professor of Industrial Pharmacy, Department of Industrial Pharmacy, College of Pharmaceutical Sciences and Drug Manufacturing, Misr University for Science and Technology (MUST), 6th of October City, Giza, Egypt; 5 Dean of Faculty of Dentistry, Professor of Laser Research Centre, Misr University for Science and Technology(MUST), 6th of October City, Giza, Egypt; 6 Professor of Oral Medicine, Department of Oral Medicine, Periodontology, Diagnosis and Radiology, Al Azhar University, Laser Research Centre, Misr University for Science and Technology(MUST), 6th of October City, Giza, Egypt

**Keywords:** Preventive dentistry, Oral diseases

## Abstract

**Introduction:**

The application of different cavity disinfectants is an essential step that eliminates bacteria after cavity preparation. However, some of these materials may affect restoration sealing ability.

**Aim:**

This study aimed to assess the degree of microleakage at the tooth restoration interface after using different nano prepared cavity disinfectants versus Diode Laser.

**Materials and methods:**

Three disinfectants were prepared on the nanoscale; Propolis, Liquorice and Chlorhexidine. A total of 40 extracted premolars with standard class V cavities were prepared on the facial surface. Teeth were divided into five groups according to the applied cavity disinfection protocol; no treatment, Chloehexidine, Propolis, Liquorice, and Diode Laser. After application of composite resin restoration, all teeth were subjected to thermocycling, afterwords the degree of microleakage was measured in micrometers. Data were analyzed by one-way analysis of variance (ANOVA) test, followed by Bonferroni’s and Tukey’s post hoc test.

**Results:**

The highest mean microlekage value was recorded in no treatment group, followed by Liquorice, Propolis. While Diode Laser group showed the lowest degree of microleakage.

**Conclusion:**

Diode Laser cavity disinfectant has no negative effect on the restoration sealing ability. Nano prepared Propolis showed comparative results to nanoChloehexidine as both had low degree of microleakage.

## Introduction

Dental caries has a high prevalence worldwide, it can be defined as a multifactorial pathology arising from the interaction between dental structure and microbial biofilm, due to the imbalance between remineralization and demineralization process, with the last one prevailing [1]. During cavity preparation, incomplete elimination of the cariogenic bacteria may lead to their survival for a long time causing secondary caries and restoration failure as well [2, 3].

Cavity disinfection has been suggested to eliminate or even reduce the microbial load just before the restorative procedure [4, 5]. Chlorhexidine (CHX) is considered the golden standard for cavity disinfection due to the antimicrobial effect and its ability of binding to bacterial^,^s amino acids [6]. Lacerda-Santos et al. [7] and Satpute et al. [8] have reported that up to 10% concentrations are acceptable to be in contact with living tissues.

Recently, there has been a growing need to replace chemical products with natural remedies as a part of dental therapeutics [9]. Propolis is a well-known natural substance extracted from bee hives with antibacterial property and others, accordingly, it is widely used in dentistry as a root canal disinfectant, mouth rinse, cavity disinfectant and others [10, 11]. Also, Liquorice has been used for different medicinal purposes, while the flavonoid component of its extract has strong antibacterial effect [11].

On the other hand, Lusche et al. [12] proved that the action of liquid antiseptics is restricted as their penetration inside the dentine is limited. Thus, it is important to evaluate depth-effective disinfection alternatives that show minimal side effects without forming bacterial resistance. Pharmaceutical scientists use various methods to enhance the dissolution and bioavailability of poorly soluble drugs such as; micronization, self-emulsification, salt formation, solid lipid nanoparticles nanocrystallization, and others [13, 14]. Awan et al. [14] stated that nanocrystallization enhances solubility and loading of the drug as well as increases its bioavailability.

Moreover, Lasers have become increasingly popular in dentistry because of their wide application. It can penetrate and kill bacteria in addition to occlusion of the dentinal tubules and thereby closing all the avenues of bacterial reinfection [15, 16]. Also, the antimicrobial effect of Diode laser significantly reduced the number of viable bacteria in the biofilm [17]. However, there are scarce data in the literature concerning the effect of Diode laser cavity disinfectant on restoration sealing ability.

Cavity disinfectants should not only have a strong antimicrobial effect but also must not compromise adhesion to the dental tissues, otherwise, it may lead to microleakage at the tooth-restoration interface [18]. Once the sealing ability is disrupted and marginal leakage occurred this consequently ends up with restoration failure [19, 20].

It was found that disinfectants may affect the adhesion of composite to dentine either positively or negatively, consequently affecting the clinical success of the restoration. Some materials have been claimed to increase the adhesion while others had a negative impact on the adhesion [5]. Moreover, there is scarce information in the literature concerning the impact of using different cavity disinfections on the degree of tooth –restoration microleakage [10].

Hence it was of interest to prepare different nanogrels; Chlorhexidine, Propolis, and Liquirose then study and compare their effect on composite microleakage versus Diode laser. The null hypothesis assumed that all of the applied cavity disinfectant protocols could not affect the sealing ability at the tooth-restoration interface negatively.

## Materials and methods

### I-Materials

See Table 1 for material information used in the study.

**Table 1:** Brand names, composition, and manufacturers of materials used in the study.

Brand name	composition	manufactures
Charisma composite	61% filler by volume, with a particle size of 0.005–10 μm. Barium, Aluminum Fluoride glass, and Pre-polymerized fillers. BIS-GMA matrix.	Kulzer, Germany.
HV Etch	37% phosphoric acid gel	Bisco, USA.
Gluma Bond Universal	Acetone/water-based solution of light-activated methacrylate monomers and Silane.	Heraeus Kulzer, Germany

### II- Methods

### Ethical approval

This in vitro study was approved by the Institutional Review Board at Misr University for Science and Technology with approval number: 2022/0066.

### Sample size calculation

Based on Bin‑Shuwaish et al. [21] using the G power statistical power. Analysis program (version 3.1.9.4) for sample size determination [22], the total sample size was 40 (8 in each group) which was sufficient to detect a large effect size (f) = 0.61, with an actual power (1-β error) of 0.8 (80%) and a significance level (α error) 0.05 (5%).

### Grouping of samples

#### Group 1

No disinfection was applied (negative control) [6].

#### Group 2

In total, 2% Chlorhexidine nanogel (positive control): The cavity was dried with an air syringe and 1 ml of the prepared gel was applied for 60 s then it was washed with saline for 30 s [2].

#### Group 3

Propolis nanogel 10% was applied for 120 s then it was washed with saline for 30 s [2, 11]

#### Group 4

Liquorice nanogel 10% was applied for 120 s then it gel was washed with saline for 30 s [11].

#### Group 5

Diode laser (Doctor Smile, Wiser, LAMBDA SpA, Italy)

Diode Laser criteria: 980 nm waves, and 1 Watt output power was applied in a continuous manner for 60 s divided into 2 cycles. Each of these cycles was 30 s using a 200 µm diameter fiber optic tip. The tip was perpendicularly directed away from the prepared cavity by 1 mm distance [2, 10].

### Preparation of samples

A total of 40 intact human premolar teeth recently extracted for orthodontic reasons were collected. All teeth were cleaned and examined under a stereomicroscope to exclude fractured tooth then they were stored until the beginning of the experiment. Standardized class V cavities (3 mm mesiodistally ×2 mm occlusogingivally ×1.5 mm buccolingually) were prepared on the facial surface of each tooth using diamond bur (HoricoDiament, Germany), 1 mm occlusal to the cementoenamel junction to assure that cavosurface line angles were prepared within enamel. A new bur was used for every five teeth with a copious amount of water coolant. Digital caliber was used to standardized the cavity dimensions [6].

All teeth were randomly divided into five experimental groups (*n* = 8) according to the cavity disinfection protocol.

### Herbal extract preparation

#### Propolis

Propolis extract was purchased from www.thehealthshop.com (Brazilian green bee Propolis, Uniflora, FDA Reg.#10760930494, Brazil).

#### Liquorice

##### Liquorice collection

Liquorice was purchased from a commercial herbal company (Al Fahd Apothecary, Egypt) and identified at the Pharmacognosy Department, Misr University for Science and Technology after permission number PG7. A voucher specimen of Liquorice under number 16.9.2020 was deposited in Pharmacognosy Laboratory, Faculty of Pharmacy, Cairo.

##### Liquorice extract preparation

In total, 200 gm of Liquorice leaves were chopped into pieces and inserted into an electric oven at 45 ± 5 °C for 24 h until complete dryness. Then it was ground using an electric miller to produce a fine powder. Each 150 gm powder was immersed in a flask containing 96% ethanol (300 mL) for 72 h at room temperature. The solution was filtered and evaporated using a rotary flask evaporator to obtain the well-concentrated extract. Finally, the extract was stored in the refrigerator at 4 °C until needed.

### Nanogel preparation

Preparation of CHX, Propolis, and Liquorice nanocrystals(NCs) at the Pharmacognosy Department, Misr University for Science and Technology:

Both CHX (C22H30CL2N10, Nerol, Egypt) and Propolis nanocrystals NCs were separately prepared through antisolvent precipitation method followed by high-shear homogenization to obtain NCs of the required size [13, 14].

To prepare the solvent phase, 2% CHX, and 10% of each of the Propolis and Liquorice extracts were first dissolved in a suitable quantity of methanol separately. The prepared solvent phase was added dropwise into the antisolvent phase with a ratio of 1:10 solvent to antisolvent containing pre-screened surfactant which is the lipid phase containing Compritol 888 ATO, Span60 solid lipid nanoparticles (4% of total concentration) with a stirring speed of 1200 rpm at room temperature [14].

Each of the prepared formulas was subjected to rotary evaporation for 20 min at 100 rpm and 70 ^◦^C to evaporate the organic phase. Then they were subjected to a homogenizer at a speed of 7000 rpm for 20 min. The developed formulations were further freeze-dried to obtain CHX, Propolis, and Liquorice Nanocrystals dried powder [23].

Each formula was assessed for entrapment efficiency percentage (EE %), Particle size (PS), Zeta potential (ZP), and Polydispersity index (PDI) (Malvern Instruments, Ltd., UK). The prepared solid lipid nanoparticles (SLNs) were incorporated into the gel using 1% noveon. The prepared nanoparticles showed particle sizes ranging from 80 to 100 nm, all with a negative charge and PDI below 0.5. The EE% ranged from 85 to 90% using Cooling Centrifuge (Sigma 3 K 30, Germany).

### Restoration

After cavity disinfection protocols, all cavities were etched using HV Etch (Bisco, USA) followed by bond application; Gluma Bond Universal (Heraeus Kulzer, Germany) according to the manufacturer’s instructions [24]. Then all cavities were restored with Charisma composite (Kulzer, Germany) and light cured according to the manufacturer’s instructions.

### Thermocycling process

All teeth were immersed in distilled water at 37 °C for 24 h. Then they were incubated for 78 s (30 s at 55 °C, 10 s stop, 30 s at 5 °C, and 8 s to return the cycle to the starting point). A total of 500 thermal cycles were conducted (TC-300 Vafai Factory Thermocycler) [25].

### Microleakage test

#### Samples preparation for dye penetration

After thermocycling, all teeth were dried and their apices were blocked with sticky wax to prevent dye penetration. Afterward, they were coated with two layers of nail varnish within approximately 2 mm of the tooth-restoration interface except for 1 mm gingivally in order to prevent dye penetration except at tooth-restoration interface [25]. Then all teeth were immersed in 2% methylene blue dye (Sparks, USA) for 24 h at room temperature. After dye penetration, teeth were rinsed and dried then sectioned longitudinally in buccolingual direction using a microtome (MTI Corporation, Richmond CA) [26].

#### Microleakage assessment

Assessment of linear dye penetration at tooth- restoration interface both occlusal and gingival was done using the stereomicroscope at 100X magnification (MA 100 Nikon stereomicroscope Japan with Omnimet image analysis software) to study the degree of microleakage in micrometers. The depth of dye penetration was measured by Omnimet image analysis software [27, 28].

### Statistical analysis

Data management and statistical analysis were performed using the Statistical Package for Social Sciences (SPSS) version 18. Data were explored for normality by checking the data distribution and using Kolmogorov-Smirnov and Shapiro-Wilk tests which revealed that most data were normally distributed (Table 2). Numerical data were summarized using mean, standard deviation, and confidence intervals. Comparisons between groups with respect to normally distributed numeric variables were compared by one-way analysis of variance (ANOVA) test, followed by Bonferroni’s and Tukey’s post hoc test. All *p* values are two-sided. *P* values ≤ 0.05 were considered significant.

**Table 2:** Tests of Normality.

	Groups	Kolmogorov-Smirnov			Shapiro-Wilk		
		Statistic	df	*P*	Statistic	df	*P*
Occlusal	No treatment	0.119	8	0.200	0.972	8	0.914
	CHX	0.220	8	0.200	0.897	8	0.272
	Propolis	0.202	8	0.200	0.908	8	0.341
	Liquorice	0.199	8	0.200	0.923	8	0.455
	Diode	0.418	8	0.000	0.528	8	0.000
Gingival	No treatment	0.209	8	0.200	0.894	8	0.256
	CHX	0.163	8	0.200	0.947	8	0.684
	Propolis	0.184	8	0.200	0.908	8	0.342
	Liquorice	0.236	8	0.200	0.865	8	0.133
	Diode	0.189	8	0.200	0.960	8	0.809
Average	No treatment	0.177	8	0.200	0.909	8	0.349
	CHX	0.215	8	0.200	0.911	8	0.365
	Propolis	0.148	8	0.200	0.941	8	0.623
	Liquorice	0.229	8	0.200	0.931	8	0.529
	Diode	0.228	8	0.200	0.903	8	0.306

*P* value > 0.05 denote normal distribution in all data except occlusal margin of diode group, *df* degree of freedom.

## Results

**Fig. 1:**
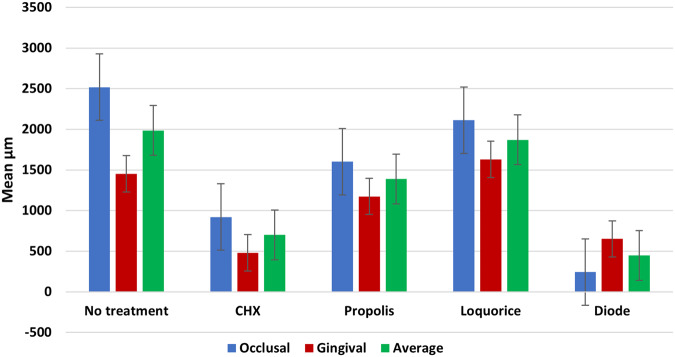
MMicroleakage. Bar chart showing mean microleakage values (µm) in different groups.

**Fig. 2:**
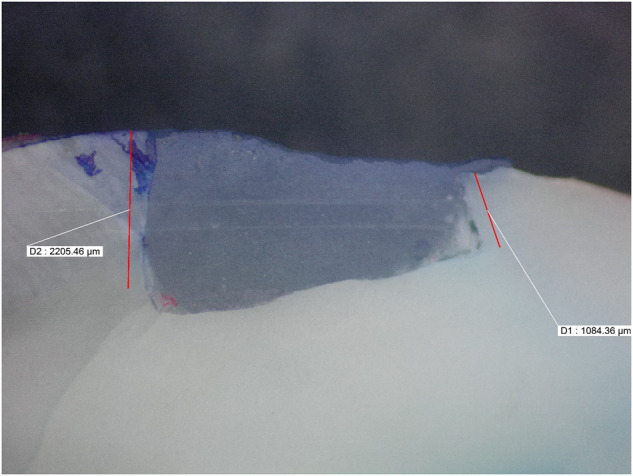
Digital image of dye penetration along tooth-restoration interface in the no treatment group. The figure shows high microleakage values at both gingival and occlusal margins. Group 1: No treatment.

**Fig. 3:**
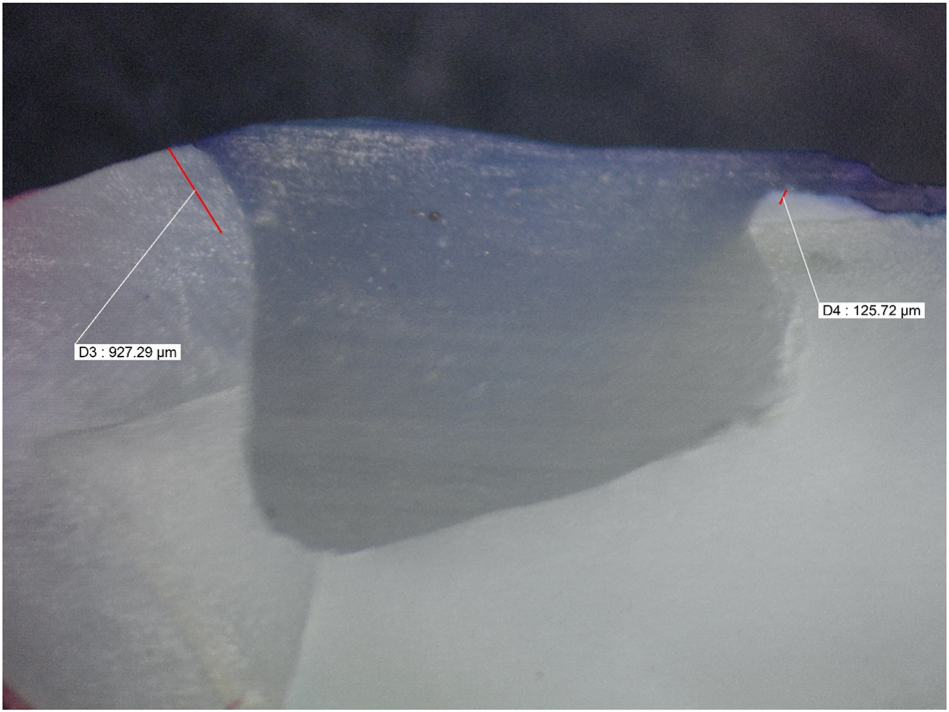
Digital image of dye penetration along tooth-restoration interface in chlorhexidine group. The figure shows low microleakage value at the occlusal margins and lesser value at gingival margin. Group 2: Chlorhexidine.

**Fig. 4:**
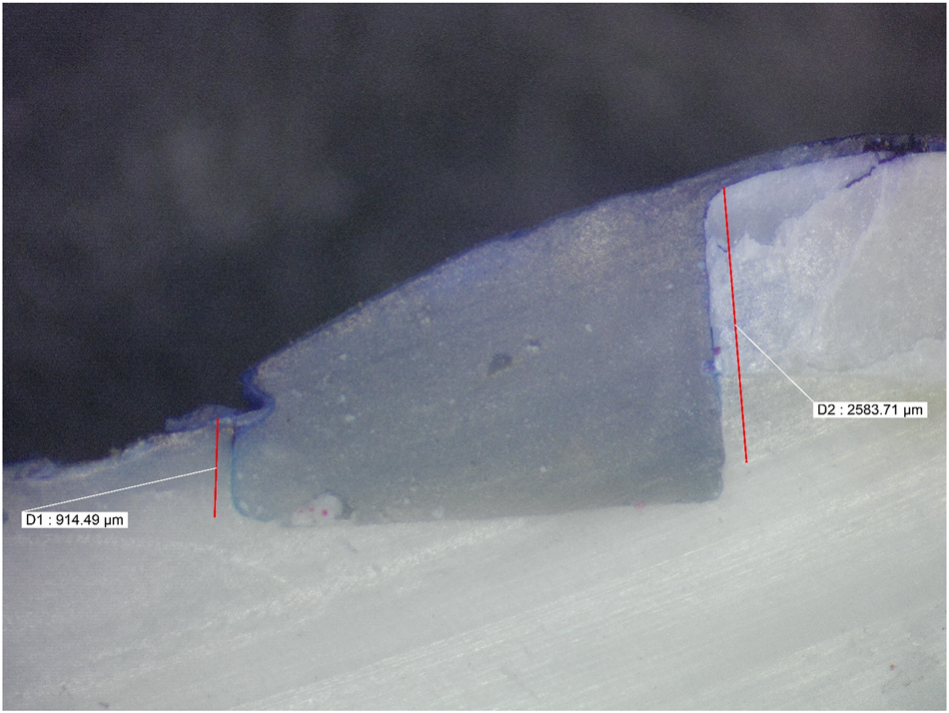
Digital image of dye penetration along tooth-restoration interface in Propolis group. The figure shows high microleakage value at the occlusal margins while the gingival one showed less microleakage value. Group 3: Propolis.

**Fig. 5:**
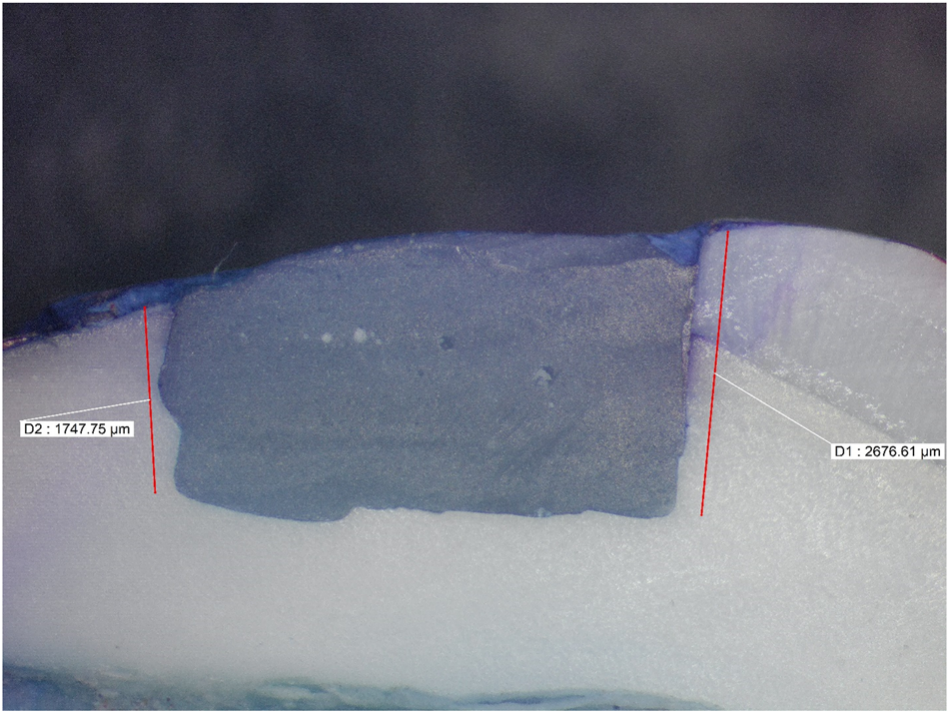
Digital image of dye penetration along tooth-restoration interface in Liquorice group. The figure shows high microleakage value at both gingival and occlusal margin. Group 4: Liquorice.

**Fig. 6:**
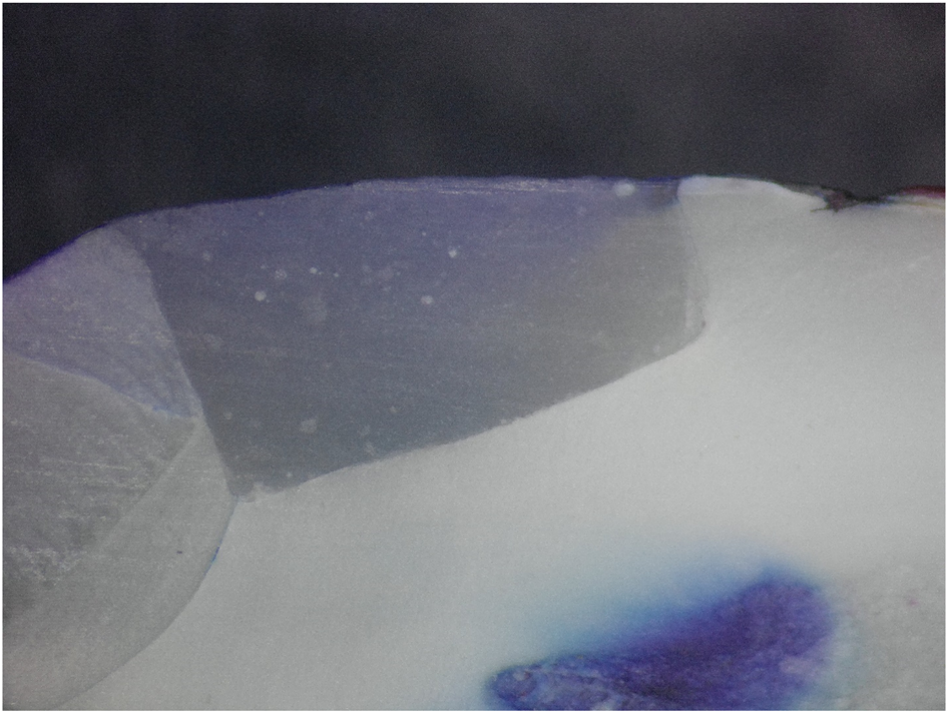
Digital image of dye penetration along tooth-restoration interface in Diode Laser group. The figure shows nearly absence of microleakage at both gingival and occlusal margins. Group 5: Diode Laser.

Microleakage results were summarized in (Tables 2, 3) and Fig. (1). Figures (2, 3, 4, 5, 6) represent digital images of dye penetration along the tooth- restoration interface.

**Table 3:** Descriptive statistics and comparison of microleakage (µm) in different groups.

		Median	Mean	Std. Dev	95% Confidence interval for mean		Min	Max	F	*P* value
					Lower bound	Upper bound				
Occlusal	No treatment	2504.50	2518.63^a^	311.79	2257.97	2779.28	2034.00	2945.00	11.354	0.000*
	CHX	923.5	920.38^b,c^	907.78	161.45	1679.30	0.00	2470.00		
	Propolis	1833.5	1600.75^a,b^	795.73	935.50	2266.00	0.00	2583.00		
	Liquorice	2216	2112.25^a^	1033.26	1248.42	2976.08	0.00	3446.00		
	Diode	0	243.88^c^	560.92	225.07	712.82	0.00	1598.00		
Gingival	No treatment	1330.5	1452.50^d^	359.34	1152.09	1752.91	1084.00	2047.00	9.061	0.000*
	CHX	417.5	479.25^f^	351.61	185.30	773.20	0.00	965.00		
	Propolis	1015.5	1174.00^d,e^	542.67	720.31	1627.69	589.00	2249.00		
	Liquorice	1703	1629.00^d^	569.07	1153.24	2104.76	391.00	2288.00		
	Diode	574	650.75^e,f^	475.41	253.29	1048.21	0.00	1392.00		
Average	No treatment	2030.25	1985.56^x^	252.39	1774.56	2196.57	1644.50	2287.00	33.785	0.000*
	CHX	613	699.81^z^	306.30	443.74	955.89	380.00	1235.00		
	Propolis	1356.5	1387.38^y^	229.80	1195.26	1579.49	1115.00	1748.50		
	Liquorice	2010.75	1870.63^x^	527.85	1429.33	2311.92	1144.00	2690.50		
	Diode	416	447.31^z^	264.42	226.25	668.37	113.50	799.00		

Significance level *p* ≤ 0.05, *significant.

Post hoc test: Within the same comparison, means sharing the same superscript letter are not significantly different.

I-At occlusal margin of the cavity:

The highest mean value was recorded in no treatment group, followed by Liquorice, Propolis and CHX groups respectively, with the lowest value recorded in Diode group. The difference between all groups was statistically significant (*p* = 0.00). Post hoc test revealed no significant difference between the no treatment, Liquorice, and Propolis groups. CHX group was not significantly different from Propolis nor from Diode groups.

II-At gingival margin of the cavity:

The highest mean value was recorded in Liquorice group, followed by no treatment, Propolis and Diode groups respectively, with the lowest value recorded in CHX group. The difference between all groups was statistically significant (*p* = 0.00). Post hoc test revealed no significant difference between the no treatment, Liquorice, and Propolis groups. Diode group was not significantly different from Propolis nor from CHX groups.

III-Average of microleakage both occlusally and gingivally:

The highest mean value was recorded in no treatment group, followed by Liquorice, Propolis and CHX groups respectively, with the lowest value recorded in Diode group. The difference between all groups was statistically significant (*p* = 0.00). Post hoc test revealed no significant difference between the no treatment group and Liquorice group. Diode group was not significantly different from CHX group.

## Discussion

After cavity preparation cariogenic microorganisms can survive in the smear layer and dentin tubules, so complete eradication of the infected tissues should be done to guarantee the durability of the final restoration. Otherwise, secondary caries can occur that leads to restoration failure [29]. Several researchers have recommended using different cavity disinfection such as different chemical agents, laser, ozone, and others [19, 29–31].

In our study, the preparation of different experimental nanaogels; Liquorice, Propolis, and Chlorohexidine was based upon several facts like being biocompatible, having high antibacterial property, and, better penetration [25, 32–35]. In addition, Wael et al. [33] stated more advantages of the hydrogels including the controlled release in addition to the high antibacterial affinity. This was coincidence with our findings where Propolis and Chlorhexidine groups showed a statistically significant low microleakage which in turn might reflect the good performance of such nanogels as they could perfectly disinfect the prepared cavities without retarding adhesion.

Concerning microleakage results as shown in (Tables 2, 3) and Figs. (2, 3, 4, 5, 6); the negative control group had high statistically significant microleakage values at both gingival and occlusal margins. This was in accordance with several studies [36–38] which proved that the negative control group had high degree of microleakage at the tooth-restoration in comparison to other groups using different cavity disinfectant protocols. On the other hand, Mutluay et al. [37] and Unal et al. [36] proved that the negative control group didn’t show any significante higher degree of microleakage than other groups using different cavity disinfectant methods. This might be due to different methodologies where different bonding technique was applied as well as using different restorative materials.

While Chlorhexidine group had low statistically significant microleakage values at the tooth restoration interface which can reach zero. This was in accordance with Ramezanian et al. [38] who proved that application of Chlorhexidine cavity disinfectant significantly reduces the degree of microleakage not only immediately but also in the long term. In addition, Satpute et al. [8] review article claimed that using Chlorhexidine enhances the durability of the restorative material due to its anti-collagenolytic action on matrix metalloproteinases enzyme (MMPs). On the other hand, Mutluay et al. [37] stated that Clorhexidine did not negatively affect microleakage in the same manner as other cavity disinfectant protocols used in their study.

Moreover, Propolis group revealed high statistically significant microleakage values in the occlusal margin while the gingival margin showed less microleakage values. This might be attributed to the medium viscosity of the prepared nanogel. Up till now, there isnt any study that evaluated the effect of Propolis cavity disinfectant on microleakage, while limited studies evaluated its antibacterial effect [9, 10, 19].

Also Liquorice group showed high statistically significant microleakage values at both gingival and occlusal margins. This finding might be attributed to the high viscosity of the prepared nanogel that could prevent further penetration of the applied bond. By consequence, this could affect composite restoration sealing ability that leads to high degree of microleakage at the tooth-restoration interface. Although Liquorice cavity disinfectant has been studied for its antibacterial effect [11] but still there isn’t any available study that evaluated its sealing ability.

On the other hand, Diode laser group 980 nm had the least statistically significant microleakage values which could reach zero-degree at gingival and occlusal margin as well. This outcome reflected the ability of Diode Laser to attain a good restoration sealing quality. Unfortunately, there was a shortage of studies evaluating the effect of Diode laser as a cavity disinfectant on the degree of microleakage at tooth- restoration interface while limited studies evaluated its effect on the bond strength or its antibacterial potentiality [10, 31, 34]. In accordance with our results, Golbari et al. [39] concluded that 810 nm Diode Laser could reduce marginal microleakage at the tooth-restorations interface. However, Ipek et al. [6] proved that the Diode laser did not show any favorable effect on the drgree of microleakage at tooth—restoration interface. This might be due to different Laser parameters used in our study, as cavities were irradiated with 2 laser cycles which might enhance the adaptation of the applied restorative material.

Thus the null hypothesis is partially rejected as CHX and Propolis nanogels as well as Diode Laser have a low degree of microleakage at the tooth-restoration interface and good sealing ability while Liquorice nanogel negatively affect the sealing ability at tooth-restoration interface.

On the other hand, some limitations were found in this study as using different types of restorative materials and bonding protocols would be of value. Also long-term studies are required to evaluate the effect of different disinfection protocols on the degree of microleakage at tooth –restoration interface.

## Conclusions

Within the limitation of this in vitro study, cavity disinfection using Propolis nannogel showed comparative results to nanoChloehexidine as both had a low degree of microleakage at the tooth restoration interface. Liquiorice nanogel had an obvious negative effect on the sealing ability of composite resin restoration while, Diode Laser didn’t affect its sealing ability.

## Benefits of the findings

- This study highlighted the importance of using either CHX nanogel orDiode laser cavity disinfectant protocols as they have either minimum or even absence of microleakage at the tooth-restoration interface.
- Propolis nanogel has a comparable effect to CHX nanogel which may be attributed to the medium viscosity of the prepared nanogel that encourages its use as a cavity disinfectant.
- Liquorice nanogel isn’t preferred as a cavity disinfectant due high degree of microleakage at the tooth-restoration interface that strongly affect the restoration sealing ability.

## Data Availability

The datasets of this study are available from the corresponding author upon reasonable request.
